# Nonlinear vibration behavior of graphene resonators and their applications in sensitive mass detection

**DOI:** 10.1186/1556-276X-7-499

**Published:** 2012-09-04

**Authors:** Mai Duc Dai, Chang-Wan Kim, Kilho Eom

**Affiliations:** 1 Department of Mechanical Engineering, Konkuk University, Seoul, 143-701, Republic of Korea; 2 Department of Biomedical Engineering, Yonsei University, Wonju, 220-740, Republic of Korea; 3 Institute for Molecular Sciences, Seoul, 120-749, Republic of Korea

**Keywords:** Graphene resonator, Mass sensing, Nonlinear oscillation, NEMS

## Abstract

Graphene has received significant attention due to its excellent mechanical properties, which has resulted in the emergence of graphene-based nano-electro-mechanical system such as nanoresonators. The nonlinear vibration of a graphene resonator and its application to mass sensing (based on nonlinear oscillation) have been poorly studied, although a graphene resonator is able to easily reach the nonlinear vibration. In this work, we have studied the nonlinear vibration of a graphene resonator driven by a geometric nonlinear effect due to an edge-clamped boundary condition using a continuum elastic model such as a plate model. We have shown that an in-plane tension can play a role in modulating the nonlinearity of a resonance for a graphene. It has been found that the detection sensitivity of a graphene resonator can be improved by using nonlinear vibration induced by an actuation force-driven geometric nonlinear effect. It is also shown that an in-plane tension can control the detection sensitivity of a graphene resonator that operates both harmonic and nonlinear oscillation regimes. Our study suggests the design principles of a graphene resonator as a mass sensor for developing a novel detection scheme using graphene-based nonlinear oscillators.

## Background

Graphene has recently been attracting the scientific community due to its excellent electrical [1-3] and/or mechanical properties [4-8]; these remarkable properties have enabled the exploitation of graphene for the development of nano-electro-mechanical system (NEMS) such as nanoresonators [9,10]. Specifically, since a pioneering work by researchers at Cornell [11], graphene has recently been extensively taken into account for designing nanoresonators that can exhibit high-frequency dynamic range [11-13] with favorable high Q factors [13-17]. The high-frequency dynamics of graphene is attributed to its excellent mechanical properties such as Young's modulus of approximately 1 TPa [4-8,18]; it is noted that a resonant frequency is linearly proportional to the square root of Young's modulus when a device operates in harmonic oscillation [9,10]. Until recently, most research works [11-15] (except a work by Eichler et al. [17]) have focused on the harmonic oscillation of a graphene resonator. However, the nonlinear vibration of a graphene resonator has not been well studied yet, albeit a recent study [17] reports an experimental observation of the nonlinear vibration of a graphene resonator. The nonlinear elastic deformation of a graphene is ubiquitous due to the fact that a monolayer graphene is an atomically thin sheet so that the out-of-plane deflection of a graphene is much larger than its thickness [19], which indicates that a graphene can easily undergo a nonlinear elastic deflection. Moreover, as discussed in our previous study [9,20,21], the nonlinear vibration is a useful route to the development of novel sensitive detection scheme based on nanoresonators made of nanomaterials such as carbon nanotubes.

To gain a detailed insight into the underlying mechanism of the vibration of a graphene resonator, an atomistic simulation such as molecular dynamics (MD) simulation has been widely utilized. For instance, Park and coworkers [22,23] have studied various effects such as edge effect and/or internal friction effect on the vibrational behavior of graphene resonators using MD simulation. Furthermore, Park and coworkers [24] have investigated the energy dissipation mechanism of vibrating polycrystalline graphenes fabricated from chemical vapor deposition method by using MD simulation. Despite the ability of MD simulation to provide detailed characteristics of the vibrational behavior of graphene resonators, MD simulation is computationally restricted to studying the vibrational behavior of a graphene resonator whose length scale is <10 nm (e.g., see refs. [22,24]). On the other hand, most experimental studies have considered a graphene resonator whose length scale is >1 μm (e.g., see refs. [11-15]). This clearly indicates that a current atomistic simulation is unable to be utilized to analyze an experimentally observed vibrational behavior of a graphene resonator whose length scale is in the order of micrometer.

The computational limitation of atomistic simulations in depicting the underpinning principles of experimentally observed mechanics of graphene resonators has led researchers [19,25-27] to consider a continuum elastic model, particularly a plate model, for unveiling the vibrational characteristics of a graphene resonator. In order for a continuum elastic model to dictate the atomistic feature of the mechanics of a graphene, the elastic constants of a continuum elastic model (e.g., plate model) for a graphene have to be determined from an atomistic simulation such as MD simulation as it was taken into account for deciding the elastic constants of atomic structures (e.g., lattice) [7,8]. Recently, a plate model with its elastic constants obtained from MD simulation has allowed revealing the mechanisms of the mechanics of a graphene. More remarkably, in a recent study by Isacsson and coworkers [19], a plate model has been utilized for studying the vibrational behavior of a graphene resonator; it is shown that the vibrational behavior of a graphene resonator predicted from a plate model, whose elastic constants were determined from atomistic model, is consistent with an experimentally observed vibration of a graphene resonator. However, a recent study by Isacsson et al. [19] has only concentrated on the harmonic oscillation of a graphene resonator, even though a graphene resonator can easily reach the nonlinear vibration regime. To the best of our knowledge, despite recent studies [28,29] theoretically reporting the nonlinear vibration of a graphene resonator, the nonlinear oscillation of a graphene resonator (particularly, nonlinearity tuning), as well as atomic mass detection using graphene-based nonlinear oscillators, has not been well studied based on a continuum elastic model and/or MD simulation.

In this work, we have studied the nonlinear vibration of a graphene resonator using a continuum elastic model, i.e., plate model. We have found that nonlinear oscillation is a useful avenue for improving the detection sensitivity of a graphene resonator and that the detection sensitivity of a graphene-based nonlinear oscillator is governed by both the actuation force (which determines the nonlinearity of vibration) and the size of a graphene resonator. It is shown that the nonlinearity of vibration for a graphene resonator can be tuned by an in-plane tension and that such in-plane tension can modulate the detection sensitivity of a graphene resonator that operates in both harmonic and nonlinear oscillations. In particular, an in-plane tension improves the dynamic frequency range and detection sensitivity of a graphene resonator that operated in harmonic oscillation, while an in-plane tension deteriorates the dynamic frequency range and sensing performance of a graphene-based nonlinear oscillator. Our study sheds light on a continuum elastic model for gaining insight into not only the underlying mechanisms of nonlinear vibration-based enhancement of the dynamic frequencies and sensing performance of a graphene resonator, but also the role of an in-plane tension in modulating the nonlinearity of a graphene resonator.

## Methods

### Theory and model

Graphene can be modeled as a plate whose mechanical deformation is attributed to the strain energy composed of bending energy *U*_B_ and stretching energy *U*_S_ represented in the form [30]

(1) $$U_{B}=\frac{1}{2}\int_{\Omega }K{\left[\nabla^{2}w\left(x,y,t\right)\right]}^{2}dxdy$$

(2) $$U_{s}=\frac{1}{2}{\int_{\Omega }\frac{E_{\text{S}}h}{4\left(1-v^{2}\right)}\left[{\left\{\partial_{\text{x}}w\left(x,y,t\right)\right\}}^{2}+{\left\{\partial_{\text{y}}w\left(x,y,t\right)\right\}}^{2}\right]}^{2}dxdy+\frac{1}{2}\int_{\Omega }N_{0}\cdot \left[\nabla w\left(x,y,t\right)\right]dxdy$$

where *κE*_S_*h*, and *ν* represent the bending rigidity, axial stretching modulus, thickness, and Poisson's ratio of a graphene, respectively, *w*(*xyt*) indicates the out-of-plane deflection of a graphene, *x* and *y* are the coordinates along the in-plane direction of a graphene, respectively, **N**_0_ is a constant axial tension (due to pre-strain) applied to a graphene, and the symbol Ω in an integrand indicates the surface integral. The strain energy can be related to a potential field prescribed to the atomic structure of a graphene, as has been elucidated in Cauchy-Born model [31-34], such as

(3) $$\sum_{i=1}^{N}U_{i}^{\text{atom}}\left(r\right)=U_{\text{B}}+U_{\text{S}}$$

Here, $U_{i}^{\text{atom}}$ is a potential field prescribed to an *i*-th carbon atom for a graphene, **r** is the atomic coordinates of a graphene, and *N* is the total number of carbon atoms for a graphene. Here, it should be noted that when the elastic constants of a graphene (i.e. *κ* and *E*_S_) are determined from Equation 2, an axial tension **N**_0_ is assumed to be zero (i.e., pre-stress is not applied to a graphene). As described in a literature [25], the force field parameters provide the elastic constants of a graphene such as *κ* = 1.5 eV and *E*_S_*h* = 2,000 eV/nm^2^.

In order to obtain the equation of motion, we need to know the kinetic energy *T* for a vibrating graphene. The kinetic energy *T* can be written as

(4) $$T=\frac{1}{2}\int_{\Omega }\rho_{0}{\left[\partial_{\text{t}}w\left(x,y,t\right)\right]}^{2}dxdy$$

where *ρ*_0_ is the mass density of a graphene; the mass density *ρ*_0_ can be straightforwardly determined from a relation of *ρ*_0_ = *Nm*_C_/*S*, where *m*_C_ is the atomic mass of a carbon atom, and *S* is the surface area of a graphene. The equation of motion for a vibrant graphene can be obtained from the minimization of a Hamiltonian *H* defined as *H* = *U* + *T* − *W*, where *W* is the work done by an external force field such as actuation force. The variation of the Hamiltonian, *δH*, can be obtained as [30,35]

(5) $$\delta H=\left[\rho_{0}\partial_{\text{t}}^{2}w+\kappa \nabla^{4}w-\frac{E_{\text{S}}}{2\left(1-\nu \right)}\left\{{\left(\partial_{x}w\right)}^{2}+{\left(\partial_{y}w\right)}^{2}\right\}\nabla^{2}w-\frac{E_{\text{S}}}{1+\nu }\partial_{x}w\partial_{y}w\partial_{\mathrm{xy}}w-N_{0}^{\alpha }\partial_{\alpha }^{2}w-f\right]\delta w=0$$

where *f* is an actuation force per unit area for a graphene, a symbol *δ* indicates a variation, *δw* is a virtual out-of-plane deflection of a graphene, a Greek index indicates the coordinates, i.e., *α* = *x* (for *α* = 1) or *y* (for *α* = 2), and a repeated Greek symbol represents Einstein's summation rule. The equation of motion is therefore given by

(6) $$\rho_{0}\partial_{\text{t}}^{2}w+\kappa \nabla^{4}w-\frac{E_{\text{S}}}{2\left(1-\nu \right)}\left\{{\left(\partial_{x}w\right)}^{2}+{\left(\partial_{y}w\right)}^{2}\right\}\nabla^{2}w-\frac{E_{\text{S}}}{1+\nu }\partial_{x}w\partial_{y}w\partial_{\mathrm{xy}}w-N_{0}^{\alpha }\partial_{\alpha }^{2}w=f$$

Here, it should be noted that in-plane displacements are ignored in the governing equation given by Equation 5 since in-plane displacements are small in comparison with out-of-plane displacement *w*(*xt*). In this work, for theoretical convenience, we assume that axial force **N**_0_ is the biaxial loading represented in the form of **N**_0_ = *N*_0_(**e**_*x*_ + **e**_*y*_), where **e**_*x*_ and **e**_*y*_ indicate the directional unit vectors in the *x* and *y* directions, respectively. Furthermore, the actuation force *f* is assumed to be in the form of *f* = *f*_0_cosΩ*t*, where *f*_0_ is the amplitude of an actuation force, and Ω is a driving frequency. For solving the equation of motion given by Equation 5, we assume that the out-of-plane displacement, i.e., *w*(*xyt*), can be decomposed in the following form [9,36-38]

(7) $$w\left(x,y,t\right)=z\left(t\right)\cdot \psi \left(x,y\right)$$

**Figure 1:**
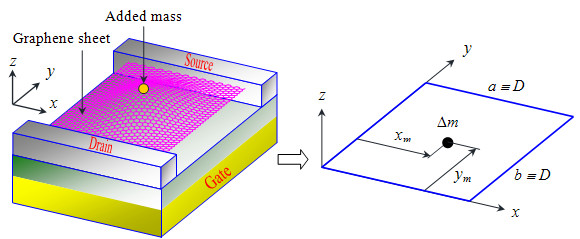
Schematic illustration of a suspended graphene resonator onto whose surface atomic mass is adsorbed.

Here, *z*(*t*) indicates a time-dependent amplitude, and *ψ*(*xy*) represents the deflection eigenmode for a vibrant graphene. In our work, we presume that a monolayer graphene exhibits a rectangular shape and that all edges of a graphene are clamped. The deflection eigenmode that satisfies the clamped boundary conditions is represented in the form

(8) $$\psi \left(x,y\right)=\frac{2}{3}\left[1-\cos\left(\frac{2\pi x}{a}\right)\right]\left[1-\cos\left(\frac{2\pi y}{b}\right)\right]$$

where *a* and *b* are the lengths of the graphene edges, respectively (see Figure 1). By substituting Equation 6 into Equation 5 followed by integration by parts, the equation of motion represented in Equation 5 becomes the Duffing equation [39-41] as follows

(9) $$\mu \partial_{t}^{2}z\left(t\right)+\alpha z\left(t\right)+\lambda {\left[z\left(t\right)\right]}^{3}=p_{0}\cos\Omega t$$

where the parameters *μαλ*, and *p*_0_ are given by

(10) $$\mu =\rho \int_{0}^{a}\int_{0}^{b}\psi^{2}\left(x,y\right)dxdy=\rho ab$$

(11) $$\begin{array}{l} \alpha =\int_{0}^{a}\int_{0}^{b}\kappa {\left[\nabla^{2}\psi \left(x,y\right)\right]}^{2}dxdy-N_{0}\int_{0}^{a}\int_{0}^{b}{\left[\nabla \psi \left(x,y\right)\right]}^{2}dxdy\\ =\frac{16\pi^{4}\kappa }{9a^{3}b^{3}}\left[3\left(a^{4}+b^{4}\right)+2a^{2}b^{2}\right]+\frac{4\pi^{2}N_{0}}{3ab}\left(a^{2}+b^{2}\right) \end{array}$$

(12) $$\begin{array}{l} \lambda =\int_{0}^{a}\int_{0}^{b}\frac{Eh}{2\left(1-\nu^{2}\right)}\psi \left(x,y\right)\left[{\left(\partial_{x}\psi \left(x,y\right)\right)}^{2}+\nu {\left(\partial_{y}\psi \left(x,y\right)\right)}^{2}\right]\partial_{x}^{2}\psi \left(x,y\right)dxdy\\ -\int_{0}^{a}\int_{0}^{b}\frac{Eh}{2\left(1-\nu^{2}\right)}\psi \left(x,y\right)\left[\nu {\left(\partial_{x}\psi \left(x,y\right)\right)}^{2}+{\left(\partial_{y}\psi \left(x,y\right)\right)}^{2}\right]\partial_{y}^{2}\psi \left(x,y\right)dxdy\\ -\int_{0}^{a}\int_{0}^{b}\frac{Eh}{1+\nu }\psi \left(x,y\right)\partial_{x}\psi \left(x,y\right)\partial_{y}\psi \left(x,y\right)\partial_{\mathrm{xy}}\psi \left(x,y\right)dxdy\\ =\frac{Ehab}{81\left(1-\nu^{2}\right)}\left[70\left\{{\left(\frac{\pi }{a}\right)}^{4}+{\left(\frac{\pi }{b}\right)}^{4}\right\}+\frac{100\pi^{4}}{a^{2}b^{2}}\left(4\nu -1\right)\right] \end{array}$$

(13) $$p_{0}=f_{0}\int_{0}^{a}\int_{0}^{b}\psi^{2}\left(x,y\right)dxdy=f_{0}ab$$

The vibrational behavior of a graphene resonator can be numerically described by solving the Duffing equation given by Equation 8.

For a case in which atoms are adsorbed onto a graphene resonator, as shown in Figure 1, we have to update a parameter *μ*, while other parameters are identical to those given by Equations 9.b to 9.d, since atomic adsorption only affects the inertia term dictated by a parameter *μ*. When atoms are locally adsorbed onto a graphene with adsorption site given as (*x*_*m*_, *y*_*m*_) as shown in Figure 1, the inertia term *μ* is given by

(14) $$\begin{array}{l} \mu =\rho ab+\int_{0}^{a}\int_{0}^{b}\Delta m\delta \left(x-x_{m}\right)\delta \left(y-y_{m}\right)\psi^{2}\left(x,y\right)dxdy\\ =\rho ab+\Delta m\psi^{2}\left(x_{m},y_{m}\right) \end{array}$$

where *δ*(*x*) is the Dirac delta function, and Δ*m* is the total mass of adsorbed atoms. For the case in which atoms are uniformly adsorbed onto a graphene (i.e., mass adsorption occurs on the entire surface of a graphene resonator), the inertia term *μ* is written as

(15) $$\begin{array}{l} \mu =\rho ab+\int_{0}^{a}\int_{0}^{b}\Delta m_{0}\psi^{2}\left(x,y\right)dxdy\\ =\rho ab+\Delta m_{0}ab\equiv \rho ab+\Delta m \end{array}$$

Here, Δ*m*_0_ is the mass of atoms adsorbed onto the unit area of a graphene, while Δ*m* is the total mass of adsorbed atoms onto the entire surface of a graphene. Based on inertia terms for both mass-adsorbed graphene (as described in Equation 10) and a bare graphene (represented in Equation 9.a), it is straightforward to compute the resonant frequency shift, Δ*ω*, of a graphene resonator due to the mass adsorption; Δ*ω* = *ω*(*m* + Δ*m*) – *ω*(*m*), where *ω*(*m*) is the resonant frequency of a bare graphene resonator whose effective mass is given as *m* (with *m* = *ρab*), and *ω*(*m* + Δ*m*) is the resonant frequency of a graphene resonator onto which the mass adsorption with the amount of Δ*m* occurs. This frequency shift due to mass adsorption is typically negative since mass adsorption increases the overall mass of a resonator and, consequently, reduces the resonant frequency of a resonator with respect to that of a bare resonator.

## Results and discussion

### Resonance behavior of graphene

**Figure 2:**
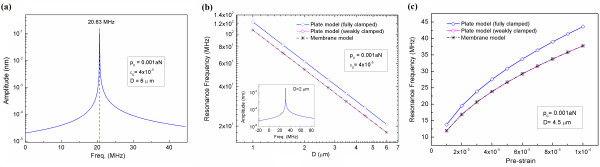
**Resonance behavior of graphene in harmonic oscillation.** (**a**) Resonance curve of a squared graphene resonator with a size of *D* = 6 μm, where a graphene operates in harmonic oscillation driven by an actuation amplitude of *p*_0_ = 10^−3^ aN. (**b**) Resonant frequencies of graphene resonators operating in harmonic oscillation as a function of not only their size but also the boundary conditions. (**c**) Resonant frequencies of a graphene as a function of pre-strain applied to it.

To verify the robustness of a continuum elastic model described in the section ‘Theory and model’, we have considered the vibration behavior of a graphene resonator with a size of 6 μm × 6 μm when a graphene is actuated by a force amplitude of *p*_0_ = 0.001 aN (where 1 aN = 10^−18^ N) in order to induce the harmonic oscillation of a graphene resonator. Moreover, we have taken into account the case in which an in-plane tension *N*_0_ is driven by pre-strain, such as *N*_0_ = *E*_*S*_*hϵ*_0_/(1 − *ν*), where *E*_S_*h*, and *ν* indicate the stretching modulus, thickness, and Poisson's ratio of a graphene resonator, respectively, and *ϵ*_0_ is the pre-strain applied to a graphene resonator. With *ϵ*_0_ = 4 × 10^−5^, the resonant frequency of a graphene undergoing a harmonic oscillation is predicted as *ω*_0_ = 20.63 MHz, which is consistent with the experimentally measured resonant frequency of 19.8 MHz (see Figure 2a and also ref. [11]). This indicates that a continuum elastic model (i.e., plate model) is suitable for understanding the dynamic behavior of a graphene resonator. It should be noted that a continuum elastic model overestimates the resonant frequency of a graphene when compared with that measured from experiments (for more details, see description as follows). 

As described above, the resonant frequency of a graphene resonator predicted from a continuum elastic model is overestimated in comparison with that measured from experiment. This may be attributed to the boundary condition that we used in our simulation; in our simulation, we have utilized the fully clamped boundary conditions such as *ψ*(*xy*) = ∇*ψ*(*xy*) = 0 along all edges; this boundary condition is referred to as “fully clamped” boundary condition. In order to understand the effect of boundary condition on the resonant frequency of a graphene resonator, we have taken into account the deflection eigenmode in the form of *ψ*(*xy*) = sin(*πx*/*a*)⋅sin(*πy*/*b*), which satisfies the boundary condition as follows: *ψ*(*xy*) = 0 at all edges, but ∇*ψ*(*xy*) ≠ 0 along all edges. This boundary condition is referred to as “weakly clamped” boundary condition. As shown in Figure 2b, the resonant frequency of a graphene resonator that is weakly clamped is almost close to the theoretical predictions obtained from the membrane model [15]. Moreover, it is shown that the resonant frequency of a graphene resonator is critically dependent on the boundary condition; the weak clamping of a graphene resonator reduces its resonant frequency. In this work, we have considered a fully clamped graphene resonator, otherwise specified. The resonant frequencies of graphene resonators predicted from our continuum elastic model (i.e., plate model) are consistent with experimentally measured frequencies of graphene resonators [15]. In addition, it is shown that pre-strain increases the resonant frequency of a graphene (Figure 2c).

**Figure 3:**
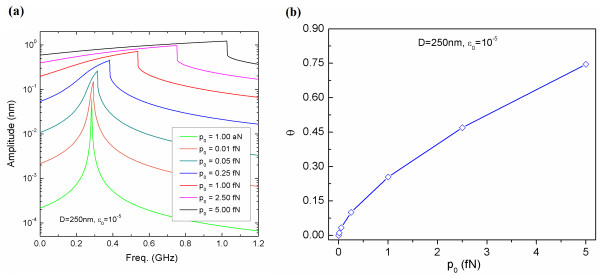
**Resonance behavior of graphene in nonlinear oscillation.** (**a**) Resonance curves of a graphene resonator (with its size of *D* = 250 nm) as a function of actuation amplitude *p*_0_. (**b**) Dimensionless parameter *θ*, which represents the degree of nonlinearity in the vibration of a graphene resonator, as a function of actuation amplitude *p*_0_.

Now, we have taken into account the nonlinear oscillation of a squared graphene resonator with a size of *D* = 250 nm, to which pre-strain with the amount of *ϵ*_0_ = 10^−5^ is applied. It is shown that when the amplitude of an actuation force is in the order of 0.01 fN, the graphene resonator undergoes harmonic oscillation. On the other hand, when a graphene resonator is actuated by the amplitude of an actuation force in the order of >0.05 fN, the graphene resonator experiences nonlinear vibration (Figure 3a). This is attributed to the geometric nonlinear effect due to fully clamped boundary condition. In order to quantitatively characterize the nonlinear vibration of a graphene resonator, we have introduced a dimensionless parameter *θ* = (*ω* − Ω_0_)/Ω_0_, where *ω* is the resonance of a nonlinearly oscillating graphene, and Ω_0_ is the harmonic resonance of a graphene defined as Ω_0_ = (*α*/*μ*)^1/2^. Here, it should be noted that the dimensionless parameter *θ* represents the degree of nonlinearity for the resonance of a graphene. Figure 3b shows the nonlinearity of graphene resonance (dictated by the dimensionless parameter *θ*) as a function of the amplitude of an actuation force. It is found that when a graphene resonator bears a pre-strain with the amount of 10^−5^, the amplitude of an actuation force in the order of 0.5 fN results in *θ* = 0.15, which indicates that the resonance behavior at an amplitude of 0.5 fN is close to the harmonic oscillation. As the amplitude increases, the dimensionless parameter *θ* significantly increases, indicating that the nonlinearity of graphene resonance can be induced by a large amplitude of actuation force. In particular, when the graphene resonator is actuated with an actuation amplitude of 5 fN, the parameter *θ* becomes *θ* = 0.75, indicating that the resonance behavior is highly nonlinear. This indicates that the nonlinear vibration of a graphene resonator can be easily observed even when the actuation amplitude is applied in the order of 1 fN. The nonlinear vibration of a graphene resonator actuated with an actuation amplitude *p*_0_ even in the order of 1 fN is attributed to the fact that the deflection amplitude of a graphene resonator is typically much larger than the thickness of a graphene resonator.

### Resonant response of graphene resonators to mass adsorption

Even though graphene resonator has recently been extensively considered as a NEMS device for its applications in actuation, it has been barely employed as a mass sensor that enables highly sensitive atomic detection (e.g., measurement of atomic weight). The high detection sensitivity of a graphene resonator is attributed to the high-frequency dynamic range that is achieved due to high elastic stiffness and low mass density of a graphene resonator. In this study, we have scrutinized the resonant responses of graphene resonators, which undergo not only harmonic oscillation but also nonlinear vibration, to atomic adsorption onto the surface of a graphene resonator. Our study on the nonlinear response of a graphene resonator to mass adsorption is ascribed to our previous finding [9,20,21] that nonlinear oscillation is useful in improving the detection sensitivity of a nanoresonator.

**Figure 4:**
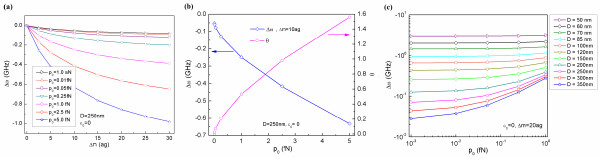
**Graphene resonators response to mass adsorption.** (**a**) Frequency shifts of a graphene resonator due to mass adsorption as a function of actuation amplitude *p*_0_ and the amount of adsorbed mass Δ*m*. (**b**) Frequency shifts of a graphene resonator due to atomic adsorption with the mass of Δ*m* = 10 ag are found as a function of actuation amplitude *p*_0_, while the dimensionless parameter *θ* of a graphene resonator with mass adsorption of Δ*m* = 10 ag is shown. (**c**) Frequency shifts of graphene resonators due to atomic adsorption with mass of Δ*m* = 20 ag with respect to the size of graphene resonators as well as the actuation amplitude *p*_0_.

We have considered a squared graphene resonator whose size is *D* = 250 nm without any applied pre-strain (i.e., *N*_0_ = 0). Figure 4a depicts the resonant frequency shift of the graphene resonator due to atomic adsorption as a function of atomic mass as well as the amplitude of an actuation force. In this work, we have assumed that atomic mass was adsorbed onto the center of a graphene resonator. In general, as shown in Additional file 1: Figure S1, the frequency shift due to mass adsorption for a graphene is dependent on the location at which atomic mass was adsorbed. Moreover, it is presumed that the stiffness of the adsorbed molecule is ignored since the elastic modulus of a graphene is in the order of 1 TPa [6], which is much higher than that of adsorbed molecules such as proteins whose elastic modulus is in the order of 10 GPa [42,43]. It is shown that the resonant frequency shift due to mass adsorption is linearly proportional to the adsorbed mass when a graphene resonator is actuated by a small amplitude of actuation force (e.g., *p*_0_ = 1 aN). On the other hand, when a graphene resonator is excited by a large amplitude of actuation force (e.g., *p*_0_ = 5 fN), the frequency shift due to mass adsorption is no longer proportional to the adsorbed mass. It is also found that as the amplitude of actuation force increases, the resonant frequency shift due to mass adsorption significantly increases, which indicates that nonlinear oscillation increases the detection sensitivity of a graphene resonator as anticipated. In order to gain a deep insight into the effect of nonlinear oscillation on the detection sensitivity of a graphene resonator, we have taken into account the resonant response of a graphene resonator to atomic adsorption with the amount of Δ*m* = 10 ag. As shown in Figure 4b, the frequency shift of a graphene resonator to atomic adsorption with the amount of Δ*m* = 10 ag is critically dependent on the amplitude of actuation force. In addition, we have shown the aforementioned dimensionless parameter *θ* as a function of the amplitude of actuation force (Figure 4b). It is shown that when the actuation amplitude is <0.5 fN, which corresponds to the harmonic oscillation (*θ* < 0.2), the frequency shift of a graphene resonator with a size of *D* = 250 nm (without any pre-strain) due to atomic adsorption with the amount of 10 ag is in the order of 0.1 GHz. On the other hand, when an actuation amplitude is increased to 5 fN, which corresponds to highly nonlinear oscillation (i.e., *θ* = 1.6), the frequency shift due to mass adsorption (with the amount of 10 ag) increases by about sixfold (i.e., Δ*ω* = 0.6 GHz). This clearly elucidates that the nonlinear vibration is useful in increasing the frequency shift of a graphene resonator due to mass adsorption, which highlights the nonlinear vibration that improves the detection sensitivity of a graphene resonator. 

We have also studied the effect of graphene size (*D*) on the frequency shift of a graphene resonator due to mass adsorption, Δ*ω*, with respect to the actuation amplitude (*p*_0_). It is found that for a graphene resonator whose size is *D* ≥ 150 nm, the increase of actuation amplitude enhances the frequency shift of a graphene resonator due to mass adsorption, which is consistent with our conjecture that nonlinear vibration improves the detection sensitivity of a graphene resonator. On the other hand, for a graphene resonator whose size is *D* ≤ 100 nm, an increase in the actuation amplitude does not significantly amplify the frequency shift of a graphene resonator due to mass adsorption in comparison to that of a large-scale graphene resonator (i.e., *D* > 200 nm). This indicates that even though large actuation amplitude induces the nonlinear oscillation of a small-scale graphene resonator (e.g., *D* < 100 nm), the nonlinear vibration does not remarkably increase the frequency shift due to mass adsorption. This result suggests that the length scale of a graphene resonator plays a central role in not only the dynamic frequency range of a graphene resonator but also in the sensing performance of graphene-based nonlinear oscillators.

### Effect of pre-strain applied to graphene resonators on their resonance behaviors and sensing performances

As described in previous studies [9,20,21], a mechanical tension (due to pre-strain or pre-stress) applied to a resonator leads to the increase of the dynamic frequency range of a resonator as well as its sensing performance. In this study, we have investigated how a pre-strain applied to a graphene resonator improves not only the dynamic behavior of a graphene resonator but also the detection sensitivity of a graphene resonator that operates in both harmonic and nonlinear oscillations.

**Figure 5:**
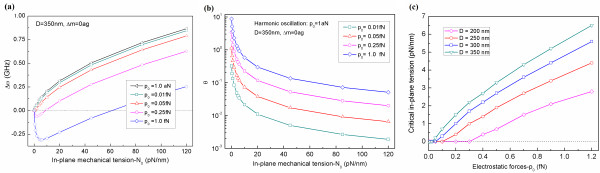
**Effects of pre-strain on bare graphene resonator.** (**a**) Frequency shifts of a bare graphene resonator (i.e. without any mass adsorption) due to in-plane tension as a function of an in-plane tension *N*_0_ and actuation amplitude *p*_0_. (**b**) Dimensionless parameter *θ* of graphene resonators as a function of actuation amplitude *p*_0_ and in-plane tension *N*_0_. (**c**) Critical in-plane tensions, which induce the transition from nonlinear vibration to harmonic oscillation for graphene resonators, are computationally obtained as a function of the size of a graphene resonator and actuation amplitude.

We have studied the frequency change of a graphene resonator due to an in-plane tension with respect to the actuation amplitude (Figure 5a). Here, the frequency change is defined as the difference between the resonant frequencies of a graphene resonator bearing an in-plane tension and a bare graphene resonator, respectively. For a graphene resonator operating in harmonic oscillation, an in-plane tension increases the resonant frequency of a graphene resonator, which attributes to the fact that an in-plane tension stiffens the system. When a graphene resonator is actuated by the actuation amplitude in the order of 1 fN (leading to the nonlinear vibration of a graphene), it is interestingly found that the application of an in-plane tension (<6 pN/nm) to a graphene resonator reduces the resonant frequency of a graphene, which indicates that the in-plane tension is not useful in increasing the dynamic frequency range of a graphene operating in nonlinear vibration. This is consistent with our previous studies [20,21] reporting that the dynamic frequency range of a nanoresonator undergoing nonlinear oscillation is decreased by a mechanical tension. However, it is remarkably found that when an in-plane tension is >6 pN/nm, the application of such in-plane tension increases the resonant frequency of a graphene resonator actuated by the amplitude of 1 fN. This may be attributed to the conjecture that an in-plane tension of 6 pN/nm to a graphene resonator actuated by an amplitude of 1 fN may induce the transition from nonlinear vibration to harmonic oscillation. 

In order to validate our conjecture that an in-plane tension can play a role in the transition from nonlinear vibration to harmonic oscillation, as shown in Figure 5b, we have plotted the dimensionless parameter *θ* (representing the degree of nonlinearity) as a function of actuation amplitude and in-plane tension. When the actuation amplitude is in the order of 0.01 fN, the vibration behavior of a graphene becomes almost harmonic oscillation regardless of in-plane tension, as anticipated. For a graphene resonator actuated by an actuation amplitude of 1 fN, the dimensionless parameter for a bare graphene resonator (i.e. *N*_0_ = 0) is in the order of 10, which indicates that the vibration behavior of a bare graphene is almost nonlinear oscillation. On the other hand, when an in-plane tension with the amount of 10 pN/nm is applied to a graphene resonator actuated by the amplitude of 1 fN, the dimensionless parameter is in the order of 0.5, which indicates that an in-plane tension reduces the nonlinearity of the resonance of a graphene. As in-plane tension increases, the nonlinearity is significantly reduced even up to the order of 10^−1^, indicating that the resonance behavior is almost harmonic oscillation. Our result suggests that an in-plane tension plays a central role not only in increasing the dynamic frequency range of a graphene resonator but also in inducing the transition from nonlinear vibration to harmonic oscillation.

Figure 5c depicts the critical in-plane tension that is responsible for the transition from nonlinear resonance to harmonic oscillation. Here, the critical in-plane tension is defined as an in-plane tension at which nonlinear oscillation is transitioned to harmonic vibration. It is found that the role of in-plane tension in such transition is highly correlated with the size of a graphene resonator. For instance, for a graphene resonator whose size is *D* = 200 nm, the resonance behavior of a graphene actuated by an amplitude of ≤0.3 fN is almost close to harmonic oscillation. On the other hand, the vibrational behavior of a 350-nm graphene resonator driven by even an amplitude of 0.1 fN becomes nonlinear oscillation. This indicates that the size of a graphene resonator determines the actuation amplitude that is required to induce the nonlinear vibration of a graphene resonator. Moreover, it is shown that the smaller the graphene resonator is, the smaller is the amount of an in-plane tension that can induce the transition from nonlinear oscillation to harmonic resonance. This suggests that an in-plane tension-driven transition from nonlinear vibration to harmonic oscillation is determined by the size of a graphene resonator.

**Figure 6:**
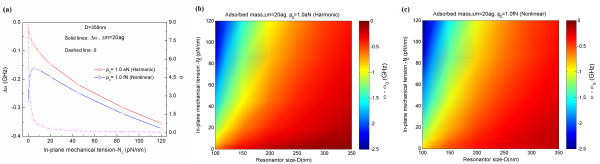
**Effects of pre-strain on graphene resonator in either harmonic or nonlinear oscillation.** (**a**) Frequency shifts of a graphene resonator, operating in either harmonic oscillation or nonlinear vibration, due to atomic adsorption with mass of Δ*m* = 20 ag as a function of in-plane tension. (**b**) Frequency shifts of graphene resonators, which undergo harmonic oscillations, due to atomic adsorption (with mass of Δ*m* = 20 ag) as a function of the size of graphene resonator and in-plane tension. (**c**) Frequency shifts of graphene resonators, operating in nonlinear vibrations, due to atomic adsorption (with mass of Δ*m* = 20 ag) with respect to the size of graphene and in-plane tension.

Now, we have studied the role of an in-plane tension on the detection sensitivity of a graphene resonator that experiences both nonlinear vibration and harmonic oscillation (Figure 6). For a graphene resonator operating in harmonic oscillation (e.g., a graphene actuated by an amplitude of 1 aN), an in-plane tension critically amplifies the frequency shift of a graphene resonator due to mass adsorption, which is consistent with our conjecture that the detection sensitivity of a graphene resonator is increased by an in-plane tension due to the in-plane tension-driven increase of the resonant frequency of a graphene. On the other hand, for a graphene resonator undergoing nonlinear vibration (e.g., actuated by an actuation amplitude of 1fN), an in-plane tension with the amount of <7 fN (corresponding to the critical in-plane tension that induces the transition from nonlinear vibration to harmonic oscillation of a graphene) decreases the amount of frequency shift due to mass adsorption, which suggests that an in-plane tension is ineffective in improving the detection sensitivity of a graphene resonator operating in nonlinear oscillation. However, when a graphene resonator is actuated by an amplitude of >7 fN, an in-plane tension increases the amount of frequency shift for a graphene resonator due to mass adsorption, which is attributed to the fact that a graphene resonator actuated by an amplitude of >7 fN obeys the harmonic oscillation. Moreover, we have also investigated the frequency shift of a graphene resonator, which operates in either harmonic oscillation or nonlinear vibration, due to mass adsorption (i.e., Δ*m* = 20 ag) as a function of the size of graphene as well as in-plane tension. It is interestingly found that for graphene resonators operating in both nonlinear oscillation and harmonic vibration, an in-plane tension-induced improvement of detection sensitivity of a graphene resonator is significantly dependent on the size of a graphene such that an in-plane tension is useful in increasing the detection sensitivity of a graphene resonator whose size is *D* ≈ 100 nm, whereas an in-plane tension is ineffective in enhancing the sensing performance of a graphene with a size of *D* > 300 nm in comparison with the detection sensitivity of a graphene resonator with a size of *D* = 100 nm. Our study sheds light on the important role of an in-plane tension on modulating not only the resonance behavior of a graphene resonator but also the detection sensitivity of a graphene resonator.

## Conclusions

In this work, we have studied the vibrational behaviors of graphene resonators as well as their sensing performance based on continuum elastic model such as plate model. It is shown that nonlinear vibration is useful in improving the detection sensitivity of a graphene resonator and that an in-plane tension is able to tune both the nonlinearity of the vibrating graphene resonators and their detection sensitivity. It should be noted that, in this work, our continuum model is only applicable to a monolayer graphene resonator. For modeling the multilayered graphene resonator, the interactions between graphene sheets have to be considered in the continuum modeling [27], which will be studied for our future work. Moreover, our continuum elastic model discards the finite size effect (i.e., edge effect) on the dynamic behavior (and also sensing performance) of a monolayer graphene resonator; here, edge (stress) effect arises from the imbalance between coordination numbers for edge atoms and bulk atoms, respectively [25,44]. This edge stress effect on a monolayer graphene is conceptually identical to the surface stress effect on a nanowire resonator [9]. Such edge effect on the frequency behavior of a graphene resonator and its sensing performance will be studied for our future work.

## Competing interests

The authors declare that they have no competing interests.

## Authors' contributions

KE and C-WK designed the research. MDD performed the simulation. MDD, C-WK, and KE analyzed the data and wrote the manuscript. All authors read and approved the final manuscript.

## Supplementary Material

Additional file 1 **Figure S1.** The dependence of frequency shift due to mass adsorption of a graphene on the location at which atomic mass was adsorbed. Click here for file
